# The EGFR/ErbB inhibitor neratinib modifies the neutrophil phosphoproteome and promotes apoptosis and clearance by airway macrophages

**DOI:** 10.3389/fimmu.2022.956991

**Published:** 2022-07-28

**Authors:** Kimberly D. Herman, Carl G. Wright, Helen M. Marriott, Sam C. McCaughran, Kieran A. Bowden, Mark O. Collins, Stephen A. Renshaw, Lynne R. Prince

**Affiliations:** ^1^ Department of Infection, Immunity and Cardiovascular Disease, The Medical School, University of Sheffield, Sheffield, United Kingdom; ^2^ Department of Infection, Immunity and Cardiovascular Disease and The Bateson Centre, The Medical School, University of Sheffield, Sheffield, United Kingdom; ^3^ Department of Biomedical Science, University of Sheffield, Sheffield, United Kingdom

**Keywords:** neutrophil, inflammation, COPD, neutrophil apoptosis, efferocytosis, mouse lung injury model, zebrafish

## Abstract

Dysregulated neutrophilic inflammation can be highly destructive in chronic inflammatory diseases due to prolonged neutrophil lifespan and continual release of histotoxic mediators in inflamed tissues. Therapeutic induction of neutrophil apoptosis, an immunologically silent form of cell death, may be beneficial in these diseases, provided that the apoptotic neutrophils are efficiently cleared from the tissue. Previous research in our group identified ErbB inhibitors as able to induce neutrophil apoptosis and reduce neutrophilic inflammation both *in vitro* and *in vivo*. Here, we extend that work using a clinical ErbB inhibitor, neratinib, which has the potential to be repurposed in inflammatory diseases. We show that neratinib reduces neutrophilic migration o an inflammatory site in zebrafish larvae. Neratinib upregulates efferocytosis and reduces the number of persisting neutrophil corpses in mouse models of acute, but not chronic, lung injury, suggesting that the drug may have therapeutic benefits in acute inflammatory settings. Phosphoproteomic analysis of human neutrophils shows that neratinib modifies the phosphorylation of proteins regulating apoptosis, migration, and efferocytosis. This work identifies a potential mechanism for neratinib in treating acute lung inflammation by upregulating the clearance of dead neutrophils and, through examination of the neutrophil phosphoproteome, provides important insights into the mechanisms by which this may be occurring.

## Introduction

Neutrophils are innate immune cells, crucial for protecting against infectious insults, and are a key cellular driver of the acute inflammatory response. If acute inflammation does not resolve, however, the continual release of inflammatory mediators such as proteases and oxidative molecules by neutrophils can be highly histotoxic and prevent tissue healing, resulting in chronic inflammation. Neutrophils are considered a major contributor to irreversible lung damage in chronic obstructive pulmonary disease (COPD), in which chronic inflammation in the lungs is induced by continual exposure to noxious substances such as cigarette smoke and pollution (1). Current treatments for COPD focus on reducing symptom severity, and none alters disease progression (2). Targeting the underlying neutrophilic inflammation may bring the much-needed new approaches for the third leading cause of death worldwide (3).

Neutrophils are one of the shortest-lived cells in the body and typically die by spontaneous apoptosis, an immunologically silent cell death mechanism in which intracellular proteins are degraded to prevent further function, but the cell membrane remains intact (4). Apoptotic neutrophils are rapidly ingested by phagocytes such as macrophages in a process called efferocytosis. If this does not occur efficiently, apoptotic neutrophils can undergo secondary necrosis, in which the cell membrane ruptures and the highly inflammatory intracellular contents spill onto the tissue, inducing further inflammation (5). Neutrophil apoptosis is known to be delayed in inflammatory environments, including in the lungs of patients with COPD during exacerbations (acute worsening of symptoms), resulting in the further release of histotoxic contents and tissue damage (6, 7). Upregulating neutrophil apoptosis may therefore be beneficial in chronic inflammatory diseases to prevent such damage; however, it would be important that these dead cells are efficiently removed from the tissue.

Previous research by our group showed that the inhibitors of the epidermal growth factor receptor (EGFR) or ErbB family of receptor tyrosine kinases are able to accelerate neutrophil apoptosis and reduce neutrophilic inflammation in several experimental models (8). Here, we have built on that work by investigating the efficacy of neratinib, a clinical ErbB inhibitor used to treat breast cancer, in mouse models of acute and chronic lung inflammation. Neratinib is known to be safe and tolerated by humans (9), making it an attractive therapeutic for repurposing.

We have also investigated the mechanism by which neratinib induces apoptosis of human neutrophils. In normal development, ErbB signaling regulates pathways controlling cellular transcription, proliferation, survival, migration, and differentiation, whereas excessive ErbB signaling in some tumor cells inhibits normal apoptosis and promotes oncogenesis (10). Pharmacological ErbB inhibitors induce tumor cell apoptosis by blocking these aberrantly activated signaling pathways; however, ErbBs are not known regulators of neutrophil apoptosis, and their role in neutrophils and other immune cells is sparsely studied. ErbBs are kinases and the regulation of their downstream signaling pathways, like many intracellular signaling networks, is controlled in part by protein phosphorylation. We therefore interrogated the “phosphoproteome” of neutrophils to obtain insights into the mechanisms by which neratinib is exerting its effects.

## Materials and methods

### Zebrafish husbandry and treatment with pharmacological agents

All zebrafish were raised and maintained according to standard protocols (11) in the UK Home Office-approved aquaria at the University of Sheffield, according to institutional guidelines. The transgenic zebrafish line *TgBAC(mpx:EGFP)i114*, in which neutrophils express the green fluorescent protein (GFP) under the myeloid-specific peroxidase (*mpx*) promoter, was used for all experiments (12, 13). For the treatment of zebrafish larvae with neratinib, larvae at 2 days post-fertilization (dpf) were immersed in E3 media containing dissolved neratinib HKI-727 (Selleck, Houston, TX, USA, S2150), or an equivalent volume of DMSO as a control, and incubated at 28°C. This was carried out in six-well plates, each well containing 6 ml of E3 and a maximum of 15 larvae. For analysis, each larva was counted as one biological replicate. Experiments were repeated three times with different batches of larvae born from different tanks of adult zebrafish.

### Whole-body neutrophil counts in zebrafish larvae

To enumerate the total number of neutrophils across the whole body of zebrafish larvae, larvae were treated for 16 h (overnight) with neratinib or DMSO, anesthetized with tricaine, then mounted in low-melting-point agarose for imaging. Images were acquired using a Nikon Eclipse TE2000 U (Nikon, Tokyo, Japan) inverted compound fluorescence microscope with NIS-Elements software. Neutrophils were enumerated manually by GFP expression, and an unpaired *t*-test was used to compare the treatment and control groups.

### Tail fin transection model of injury-induced inflammation in zebrafish larvae

To assess neutrophil number at the site of inflammation, a tail fin transection model of injury-induced inflammation was used. After 16 h of treatment with neratinib or DMSO, larvae were anesthetized using tricaine, and complete transection of the tail fin was carried out using a sterile scalpel as described previously (13, 14), at a site distally adjacent to the circulatory loop. After transection, larvae were transferred to fresh E3 containing neratinib or DMSO. At 4 and 8 h post-injury, larvae were anesthetized with tricaine and neutrophils at the injury site enumerated based on GFP expression, using either a Leica Fluorescence Stereo Dissecting Microscope (Leica, Wetzlar, Germany) or a Nikon Eclipse TE2000 U with NIS-Elements software. Two-way ANOVA with Sidak’s multiple comparisons was used to compare treatment groups at each timepoint.

### Isolation of neutrophils from human whole blood

*In-vitro* experiments were conducted on neutrophils isolated from peripheral blood samples of healthy donors, in compliance with the guidelines of the South Sheffield Research Ethics Committee (reference number STH13927). The volume of blood required was calculated prior to collection according to experimental requirements, based on approximately 2 million isolated neutrophils per 1 ml of blood. Peripheral blood samples were obtained by venipuncture and collected into a 50-ml syringe, then immediately transferred into a 50-ml Falcon tube containing EDTA, 122 μl per 10 ml of blood.

Isolation of neutrophils was carried out using the EasySep™ Direct Human Neutrophil Isolation Kit (Stem Cell Technologies, Vancouver, Canada, 19666), an immunomagnetic negative selection kit, as per the kit instructions. In brief, the antibody Isolation Cocktail and magnetic RapidSpheres (25 μl each per 1 ml of blood) were added to the blood sample and incubated for 5 min, and the tube was then placed in the EasySep magnet for 5 min. After incubation, the enriched neutrophil sample was removed and transferred to a fresh tube, leaving non-neutrophils bound to the antibody/bead complex immobilized against the side of the original tube by the magnet. Two more magnet incubations of the enriched neutrophil sample were carried out to further purify the neutrophils. The final purified neutrophil sample was washed by centrifugation at 300*g* for 6 min and resuspended in 10 ml of phosphate-buffered saline (PBS). A hemocytometer counting chamber was used to conduct cell counts, and the washed neutrophils were resuspended in RPMI 1640 media (Lonza, Basel, Switzerland, BE12-702F), supplemented with 10% fetal bovine serum (FBS) (Gibco, Waltham, MA, USA, 10500-064) at a concentration of 5 × 10^6^ cells/ml.

### Treatment of human neutrophils with pharmacological inhibitors and assessment of apoptosis

Isolated human neutrophils were added to 96-well flexible untreated polyvinyl chloride general assay plates (Corning, New York, USA, CLS2592), which minimize neutrophil adherence and activation. The plates were prepared in advance with the addition of neutrophils, with each well containing 50 μl of neratinib HKI-272 (Selleck, S2150), EX527 (SIRT1 inhibitor—Santa Cruz Biotechnology, Dallas, TX, USA), NSC348884 (NPM1 inhibitor—Selleck), or DMSO at 2× desired concentration in RPMI 1640 + 10% FBS media. Two technical replicates (wells) of each drug and concentration were generated. Immediately after isolation, 50 μl of neutrophils at a concentration of 5 × 10^6^/ml were added to each well, diluting the pharmacological agent to the desired concentration. Plates were incubated at 37°C in 5% CO_2_ for 6 h.

For the fixation, staining, and imaging of neutrophils, cytocentrifugation was used to transfer a monolayer of cells onto glass microscopy slides, referred to as “cytospin slides.” Cells in each well were transferred to a cytospin funnel secured with a clamp onto a glass microscope slide and centrifuged in a Shandon Cytospin 4 cytocentrifuge (Thermo Electron Corporation, Waltham, MA, USA) at 300 rpm for 3 min. Neutrophils (now immobilized on the microscope slide) were fixed with a drop of 100% methanol and stained by submersion in Kwik-Diff Solution 2 (Thermo Electron Corporation, 9990706) followed by Kwik-Diff Solution 3 (Thermo Electron Corporation, 9990707). Excess stain was washed from the slide using tap water, and once dry, a drop of DPX (Sigma-Aldrich, St. Louis, MO, USA, 44581) was added directly onto the cells, and a coverslip was placed carefully on top. The slides were left in the fume hood for at least 24 h to allow the DPX to set, then imaged using a Nikon Eclipse TE300 inverted light microscope with ×100 magnification oil immersion lens. Apoptotic neutrophils were identified based on nuclear morphology, which in apoptotic neutrophils is rounded and condensed, in comparison to the distinctive multilobed nuclei of healthy neutrophils. To calculate the percentage apoptosis, 300 neutrophils per cytospin slide were counted and recorded as healthy or apoptotic. As two technical replicates were generated per condition, a mean percentage apoptosis was calculated to generate one data point per condition per blood donor. Data were analyzed using a one-way ANOVA with Dunnett’s multiple comparisons, comparing each neratinib concentration to the DMSO (control)-treated group.

### Mass spectrometry-based phosphoproteomic analysis of human neutrophils treated with neratinib

Neutrophils were isolated from the blood of five healthy volunteers, as described above. Neutrophils from each donor were split into two samples of 15 million cells each and treated with 25 μM of neratinib (Sellek) or equivalent concentration (v/v) of DMSO and incubated for 1 h at 37°C and 5% CO_2_. All samples were then treated with 500 μM of db-cAMP (Sigma) for 30 min in the same incubation conditions. After incubation, the samples were centrifuged at 400*g* for 3 min at 4°C, and the cell pellet was resuspended in 1 ml of ice-cold PBS. Cells were centrifuged again at 400*g* for 3 min at 4°C, the PBS supernatant was removed, and cell pellets were stored immediately at −80°C.

To extract protein from the samples, the following lysis buffer was added to the neutrophil pellets: 5% SDS, 50 mM of TEAB buffer, 50 mM of sodium fluoride, 50 mM of β-glycerophosphate, 10 mM of sodium orthovanadate, 1 mM of PMSF, and 5% Protease Inhibitor Cocktail Set III (Calbiochem, San Diego, CA, USA, 535140) made up in HPLC-grade water. DNA was sheared from the lysed cells using a homogenizer. The samples were incubated at 70°C for 15 min, homogenized again, and incubated for a further 5 min, or until no cellular material was visible. Samples were centrifuged at 16,000*g* to pellet any cell debris, and the protein supernatants were transferred to fresh Eppendorf tubes. The samples were reduced with 10 mM of TCEP and alkylated with iodoacetamide.

Protein purification was carried out using suspension trapping (S-Trap™) columns which contain a protein-binding matrix (Protifi, Long Island, NY, USA, K02-Micro-10). To each 120 μl of protein extract, 12 μl of 12% phosphoric acid and 840 μl of binding buffer (90% methanol, 100 mM of TEAB buffer) were added. The samples were transferred to the top chamber of an S-Trap™ spin column and centrifuged at 4,000*g* for 15 s. The proteins (now “trapped” in the matrix) were washed four times with 400 μl of binding buffer and centrifuged as before to elute any impurities. The proteins were digested into peptides with 12 μl of 1:10 Trypsin Gold (Promega, Madison, WI, USA, V528A) in 50 mM of TEAB per sample and incubated at 47°C for 1 h. The peptides were then eluted from the S-Trap™ matrix with 80 μl of 50 mM TEAB and centrifuged at 1,000*g* for 30 s, followed by 80 μl of 0.2% formic acid solution and centrifuged again at 1,000*g* for 30 s. The peptides were desalted using Sep-Pak^®^ Light C18 Cartridge (Waters, Wilmslow, UK, WAT023501) and dried down using a SpeedVac (Thermo Scientific, Waltham, Massachusetts, USA).

Immobilized metal affinity chromatography (IMAC) was used to enrich phosphorylated peptides, using MagReSyn^®^ Ti-IMAC beads (ReSyn Biosciences, Gauteng, South Africa, MR-THP002). The peptides were firstly resuspended in IMAC loading buffer (1 M of glycolic acid, 80% acetonitrile, 5% trifluoroacetic acid) and centrifuged at 1,000 rpm for 5 min. Beads were placed on a magnetic rack and washed with IMAC loading buffer, after which the peptide samples were added to the beads and incubated for 20 min. After centrifugation at 1,000 rpm, the supernatant was removed and three washes were carried out using 100 μl of IMAC loading buffer per wash. The enriched phosphorylated peptides were eluted from the beads with 80 μl of 1% ammonia, then acidified with 40 μl of 10% trifluoroacetic acid.

Phosphopeptides were analyzed by high-performance liquid chromatography–mass spectrometry, using the HPLC column Acclaim^®^ PepMap 100 C18 nano/capillary BioLC (Thermo Fisher Scientific, 164535) and EASY-Spray column (Thermo Fisher Scientific, ES803), and also analyzed on an Orbitrap Elite™ Hybrid Ion Trap. Raw data were analyzed using MaxQuant version 1.6.10.43 software. The peptide spectra were searched against a human UniProt fasta file (downloaded May 2019) using the following search parameters: digestion set to trypsin/P with a maximum of 2 missed cleavages, oxidation (M), N‐terminal protein acetylation and phosphorylation (STY) as variable modifications, cysteine carbamidomethylation as a fixed modification, match between runs enabled with a match time window of 0.7 min and a 20‐min alignment time window, label‐free quantification enabled with a minimum ratio count of 2, minimum number of neighbors of 3, and an average number of neighbors of 6. A first search precursor tolerance of 20 ppm and a main search precursor tolerance of 4.5 ppm were used for FTMS scans and a 0.5-Da tolerance for ITMS scans. A protein false discovery rate (FDR) of 0.01 and a peptide FDR of 0.01 were used for identification level cutoffs and an FLR of 5% was used for phosphosite localization. Statistical analysis of the phosphorylation site data was performed using Perseus version 1.6.10.50. Phosphorylation site intensities were transformed by log2(*x*), normalized by subtraction of the median value, and individual intensity columns were grouped by experiment. Phosphorylation sites were filtered to keep only those with a minimum of 3 valid values in at least one group. The distribution of intensities was checked to ensure standard distribution for each replicate. Missing values were randomly imputed with a width of 0.3 and a downshift of 1.8 from the standard deviation. To identify significant differences between groups, two-sided Student’s *t*-tests were performed with a permutation-based FDR of 0.05.

In addition to the statistical analysis, phosphorylated proteins were also considered of interest if they were enriched in one treatment group. Proteins of interest were further analyzed using the online tools STRING and Reactome, which identify interactions between proteins. Both tools also identify functionally enriched biological processes (extracted from Gene Ontology), i.e., processes that are observed within these networks more frequently than expected based on hypergeometric testing (15–17).

### Mouse husbandry and models of lung inflammation

Approval for working with mammalian models was authorized by the Home Office under the Animals (Scientific Procedures) Act 1986 under project license P4802B8AC held by HM and personal licenses (PIL) held by CW, SM, and HM and reviewed by the Animal Welfare and Ethical Review Body at the University of Sheffield. All housing parameters conformed to the Code of Practice for the housing and care of animals bred, supplied, or used for scientific procedures. Food (Teklad 2018, Envigo, Indianapolis, IN, USA) and water were given *ad libitum*, and animals were kept on a 12-h light–dark cycle. To adhere to humane endpoints, if mice appeared in distress and were not comfortably breathing 24 h after a procedure, or if 20% weight loss was reached, mice were culled to prevent excessive suffering. Female C57BL/6J mice, aged 9–12 weeks and weighing 16–20 g at the start of each study, were used for all the experiments.

In the acute lung injury study, 12 mice were anesthetized with gaseous isoflurane and administered 7 μg of lipopolysaccharides (LPS) from *Escherichia coli* O26:B6 (LPS—Sigma, L8274) in 50 μl of PBS. Immediately after, six mice were administered 200 μl of vehicle (0.5% methylcellulose + 0.4% Tween-80 + 1% DMSO) by oral gavage, and the remaining six mice were administered 20 mg/kg of neratinib (ApexBio Technology, Houston, TX, USA, A8322) dissolved in vehicle, by oral gavage. Mice were given immediate heat support and observed regularly by the experienced PIL holders. After 48 h, all mice were sacrificed by terminal anesthesia, by intraperitoneal administration of 100 μl of pentobarbitone (100 mg/ml), and subjected to bronchoalveolar lavage (3× 1 ml administrations of PBS).

In the two chronic lung injury studies, 16 mice per study were administered 7 μg of LPS (as above) combined with 1.2 U of porcine pancreatic elastase (Merck, Darmstadt, Germany, 324682) in 50 μl of PBS, by intranasal delivery as above. This was carried out weekly for 4 weeks, i.e., on days 0, 7, 14, and 21. For the induction dosing study, neratinib or vehicle was administered to the mice as above (eight mice per treatment group), immediately after each LPS/elastase administration. This protocol is known to result in COPD-like features within 4 weeks, including sustained neutrophilic inflammation, increased alveolar macrophage numbers, airway hyperresponsiveness, and emphysema after 2 weeks (18). For the therapeutic dosing model, neratinib or vehicle doses were given on days 14, 15, 16, 17, 18, 21, 22, 23, 24, and 25. In the induction dosing study, three mice were culled prior to the end of the study due to weight loss (two in the vehicle treatment group and one in the neratinib treatment group). Two mice were culled in the therapeutic dosing study prior to the end for the same reason: one from the vehicle treatment group and one from the neratinib treatment group. In both chronic disease models, all remaining mice were sacrificed by terminal anesthesia on day 28, and bronchoalveolar lavage fluid was collected along with blood samples by inferior vena cava bleed.

### Preparation of mouse bronchoalveolar lavage samples for cell counting, microscopy, and enzyme-linked immunosorbent assay

Cells in bronchoalveolar lavage (BAL) samples were placed immediately on ice after collection. A hemocytometer counting chamber was utilized to calculate the total number of cells in each sample. In a centrifuge pre-cooled to 4°C, BAL samples were centrifuged at 400*g* for 5 min to pellet the cells, and the supernatant was removed and stored immediately at −80°C for later analysis by enzyme-linked immunosorbent assay (ELISA). The remaining cells were resuspended in ice-cold PBS at a concentration of 2 million/ml. Cytospin funnels were assembled, and 50 μl of each BAL sample was transferred to each funnel, to which 50 μl of FBS was added to prevent the cells from breaking during centrifugation. A cytocentrifuge was used to transfer the cells to microscope slides, and the slides were fixed and stained with Kwik-Diff as described above for human neutrophils.

### Preparation of mouse bronchoalveolar lavage for flow cytometry

The cells remaining in each BAL sample were prepared for flow cytometry. For all mouse studies, cells were stained with FITC anti-mouse Ly6G/Ly6C antibody (BioLegend, San Diego, CA, USA, 108405) to detect neutrophils, with PE-Annexin V (BioLegend, 640908) which binds to apoptotic cells, and with TO-PRO™-3 Iodide (Invitrogen, Waltham, MA, USA, T3605), a vital dye that only binds to dead cells (also referred to as “corpses”) in which the plasma membrane is broken. All samples were stained with all three markers, and an additional four samples were generated from the BAL samples with the most cells: one unstained control and three single-stain controls. All samples were centrifuged at 400*g* for 3 min at 4°C to pellet the cells and resuspended in 50 μl of FITC-Ly6G staining solution diluted 1:200 in FACS buffer (PBS + 10% FBS) or 50 μl of FACS buffer for controls. The samples were incubated on ice in the dark for 20 min and centrifuged at 400*g* for 3 min at room temperature and resuspended 50 μl in PE-Annexin V antibody diluted 1:20 with Annexin V binding buffer (BioLegend, 422201) or 50 μl of Annexin V binding buffer only for controls. The samples were incubated in the dark at room temperature for 20 min, after which 5 μl of TO-PRO-3 solution (diluted 1:1,000 in Annexin binding buffer) was added to each sample. The samples were kept in the dark at room temperature while the flow cytometry analyzer was set up (approximately 10 min). When the samples were ready for analysis, 300 μl of Annexin binding buffer was added to each sample to ensure an adequate volume for analysis. The samples were run on a BD LSRII Flow Cytometer (BD Biosciences, Franklin Lakes, NJ, USA) using the BD FACSDiva™ software, and data analysis was carried out using the FlowJo software (BD Biosciences).

### Analysis of mouse blood samples using an automated hematology analyzer and preparation of plasma for ELISA

Blood was collected into EDTA-coated tubes to prevent clotting and mixed gently. The majority of each sample was transferred to a freshly labeled Eppendorf tube, leaving 50 μl of blood in each EDTA tube for analysis by an automated hematology blood analyzer. Blood samples from the first chronic lung disease mouse study (induction neratinib dosing) were analyzed using Sysmex KX-21N™ (Hyogo, Japan), which had been adapted in-house to measure parameters for mouse blood cells, including leukocyte concentration, and for differential detection of neutrophils, monocytes, and lymphocytes. For the second chronic lung disease study (therapeutic neratinib dosing), blood samples were analyzed using a scil Vet abc Plus^+^ (Scil, Viernheim, Germany) automated hematology analyzer, which is programmed to analyze blood samples from a range of animals including mice.

The remaining blood samples were centrifuged at 350*g* for 10 min at 4°C to separate the plasma from the blood cells. The upper plasma layer was transferred to a fresh Eppendorf tube and stored immediately at −80°C for later analysis by ELISA.

### IL-6 and CXCL1/KC ELISA

The ELISA kit was used to determine the concentration of cytokines in bronchoalveolar lavage fluid and plasma samples from mice. ELISA kits used were mouse IL-6 DuoSet ELISA (R&D Systems, Minneapolis, MN, USA, DY406-05) and mouse CXCL1/KC DuoSet ELISA (R&D Systems, DY453-05), and the assays were carried out as per the kit instructions. All BAL samples were run neat. Two technical replicates of all samples and standards were used. Plates were analyzed on a Thermo Scientific Varioskan^®^ Flash microplate reader at 450 nm, with wavelength correction at 540 nm. Cytokine concentration was calculated using interpolation of a four-parameter logistic sigmoidal standard curve, as suggested by the kit instructions.

### Histological analysis of mouse lung tissue

The lungs were dissected upon culling and fixed in 4% paraformaldehyde for at least 48 h, before being transferred to PBS. The lungs were placed into a tissue cassette and embedded in paraffin wax, and 5-μm sections were generated and transferred to microscope slides using a microtome. Sections were stained with hematoxylin (Sigma, H3136) and eosin (Sigma, HT110116) (H&E), by moving slides sequentially through a series of reagents, spending 1–2 min in each reagent: xylene, 100% ethanol, 90% ethanol, water, hematoxylin, water, 95% ethanol, eosin, 95% ethanol, 100% ethanol, and xylene.

Histology slides were analyzed on a Nikon Eclipse E600 using either a ×10 or ×20 objective lens, and images were obtained using the NIS-Elements software. Alveoli were identified by an airspace (lumen) surrounded by characteristically thin squamous epithelial cells (pneumocytes). These were distinguishable from bronchioles, which have a thicker wall of oblong epithelial cells. Within the NIS-Elements, the polygon tool was used to manually trace the edges of individual alveoli. This tool generates a perimeter and area measurement for each shape, which were exported for analysis. Alveoli were classified as intact or damaged. Twenty alveoli per mouse, across two sections of lung tissue, were measured for analysis.

### Statistical analysis of data from mouse studies

Statistical analysis on the data collected from mouse studies compared the vehicle and neratinib treatment groups. Datasets contained a single data point for each mouse, which in some cases were calculated as an average of technical replicates or multiple measurements (e.g., ELISA, histology), but others were from a single measurement (e.g., leukocyte number in blood samples). An unpaired *t*-test was used to compare neratinib vs. vehicle treatment groups.

## Results

### Neratinib treatment reduces neutrophilic inflammation in a larval zebrafish injury model and induces apoptosis of human neutrophils *in vitro*

**Figure 1:**
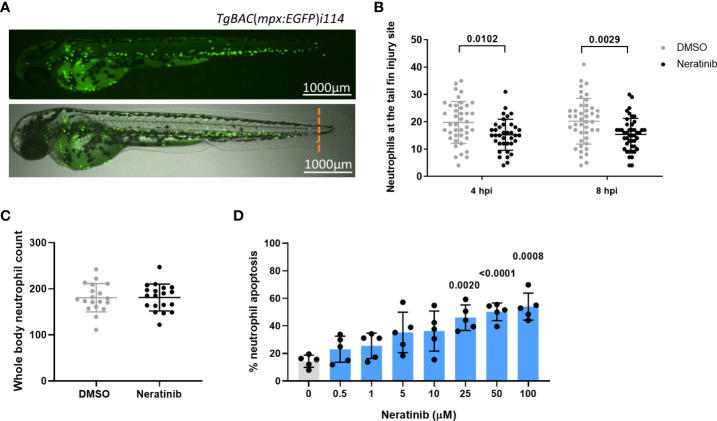
Neratinib reduces neutrophilic inflammation at the tail fin injury site of zebrafish larvae *in vivo* and induces apoptosis of human neutrophils *in vitro*. Neutrophils in zebrafish larvae can be enumerated by fluorescence microscopy using the transgenic line *TgBAC*(*mpx:EGFP*)*i114*, in which each GFP-expressing (green) cell is counted as one neutrophil **(A)**. Tail fin transection (A, orange dotted line) was performed after 16 h of treatment with neratinib, and neutrophils at the site of injury enumerated 4 and 8 h post-injury. Larvae treated with neratinib had reduced numbers of neutrophils at the tail fin injury site at both time points in comparison to DMSO-treated larvae **(B)**. Minimum *n* = 30 larvae per condition across three experimental repeats; data analyzed by two-way ANOVA with Sidak’s multiple comparisons. Total neutrophil number across the whole body of larvae was unchanged after 16 h of treatment with 10 μM of neratinib, in comparison to control DMSO-treated larvae **(C)**. *N* = 20 larvae per condition across three independent experiments; data analyzed by unpaired *t*-test. In human neutrophils isolated from whole-blood samples from healthy volunteers, treatment with neratinib *in vitro* results in a dose-dependent increase in the rate of apoptosis **(D)**. *N* = 5 healthy blood donors; data analyzed by one-way ANOVA with Dunnett’s multiple comparisons, comparing each neratinib concentration with the DMSO control treatment. Each data point represents data from one larva or human blood donor. Bars show mean ± standard deviation. *p*-values are indicated, where *p* <0.05.

Research in our previous paper used a larval zebrafish model of injury-induced inflammation to test the effect of ErbB inhibitors on neutrophils in an inflammatory environment. We showed that treatment of larvae with the research-grade ErbB inhibitors tyrphostin AG825 and CP-724,714 reduced neutrophil number at the tail fin injury site (8). Here, we tested the clinical ErbB inhibitor, neratinib, to determine if it is similarly effective at reducing neutrophilic inflammation in this model. Using the transgenic zebrafish neutrophil reporter line *TgBAC(mpx:EGFP)i114* (**Figure 1A**), we found that 16 h of pre-treatment of larvae with 10 μM of neratinib reduced the number of neutrophils at the injury site at both 4 and 8 h post-injury (**Figure 1B**). We also found that the total number of neutrophils across the whole body of zebrafish larvae was unchanged with neratinib treatment, suggesting that neratinib is acting specifically on the neutrophilic response to inflammation (**Figure 1C**).

We have previously shown that a number of ErbB inhibitors accelerate the rate of apoptosis of human neutrophils *in vitro*, and here, we confirm this using neratinib. Neratinib treatment was shown to induce apoptosis after 6 h of treatment, reaching significance at 25 μM (**Figure 1D**). These findings show that this clinical ErbB inhibitor also promotes human neutrophil apoptosis *in vitro*, at similar concentration ranges as research-grade ErbB inhibitors.

### Phosphoproteomic analysis of human neutrophils reveals potential mechanisms of action for neratinib

Although ErbB signaling has been studied widely in areas such as development and cancer, the details of these signaling pathways in neutrophils, or any other immune cells, are sparse in the literature. Since neutrophils are one of the shortest-lived cells in the human body and rapidly undergo spontaneous apoptosis, we considered that the mechanism by which neratinib induces neutrophil apoptosis may be different to that described in other cell types. As protein phosphorylation is the key event downstream of receptor tyrosine kinases and regulates many cellular processes, we investigated this using mass spectrometry-based differential phosphoproteomics to identify changes in the neutrophil phosphoproteome that occur with neratinib treatment.

For this experiment, neutrophils were isolated from the blood of five healthy volunteers and treated with either 25 μM of neratinib or an equivalent volume of DMSO (control) for 1 h, followed by a cell-permeable analog of cyclic AMP, dibutyryl-cAMP (db-cAMP), for 30 min. Cyclic AMP suppresses apoptosis in human neutrophils (8, 19), switching on pro-survival signaling pathways that, based on previous findings, we hypothesize will be suppressed in neratinib-treated cells. Neutrophil proteins were isolated and digested, and enriched phosphopeptides were identified and quantified using LC-MS/MS analysis. A single DMSO-treated sample was excluded from analysis due to poor correlation with other samples.

Details of all phosphopeptides detected, including information about specific phosphorylation events, are detailed in **Supplementary File 1**. Since all samples were treated with db-cAMP, it was expected that phosphorylated peptides mapping to proteins downstream of cAMP signaling pathways would be detected. Protein kinase A is directly activated by cAMP, and several phosphopeptides mapping to subunits of this protein complex were identified in the dataset (**Supplementary Figure 1A**). Other phosphopeptides mapping to downstream proteins present in the dataset included BRAF, CREB, GSK3A, and several MAPK family members. The detection of these phosphorylated peptides in the neutrophil samples was considered an effective validation of the dataset.

To determine if neratinib was modifying the phosphorylation of proteins within the ErbB signaling pathways described in literature, we interrogated the dataset for downstream components of ErbB signaling as above. A key downstream mediator of ErbB signaling is the PI3K family. Phosphopeptides mapping to several different subunits of this family were identified (PIK3AP1, PIK3R1, PIK3R5), which were detected in similar numbers of samples in both treatment groups (**Supplementary Figure 1B**). Similar results were observed for members of the AKT family and several members of the MAPK family. Although there are differences in the detection of some phosphopeptides between the treatment groups, such as STAT3 which was detected in two out of five neratinib-treated samples and all DMSO-treated samples, the majority of phosphorylated proteins downstream of ErbB signaling were not differentially detected (**Supplementary Figure 1B**), suggesting that neratinib is not modifying ErbB signaling pathways that are described in the literature.

We then used an unbiased approach to identify differences in the phosphoproteome of neratinib- vs. DMSO-treated neutrophils. Statistical analysis was used to determine if the abundance of any phosphopeptide was different between the two treatment groups. At 5% false discovery rate, 16 phosphorylated peptides mapping to 15 proteins were identified as statistically regulated (**Supplementary File 1**, statistical analysis tabs), with 9 phosphopeptides mapping to 8 proteins statistically increased in the DMSO treatment group and 7 in the neratinib treatment group (**Supplementary Figure 2A**). This dataset of 15 proteins was input into the online tool STRING, which identifies interactions between proteins. One interaction was identified between MAML1 and NCOR1, which were both more abundant in neratinib-treated samples (**Supplementary Figure 2B**); however, no other proteins in this dataset were found to interact.

**Figure 2:**
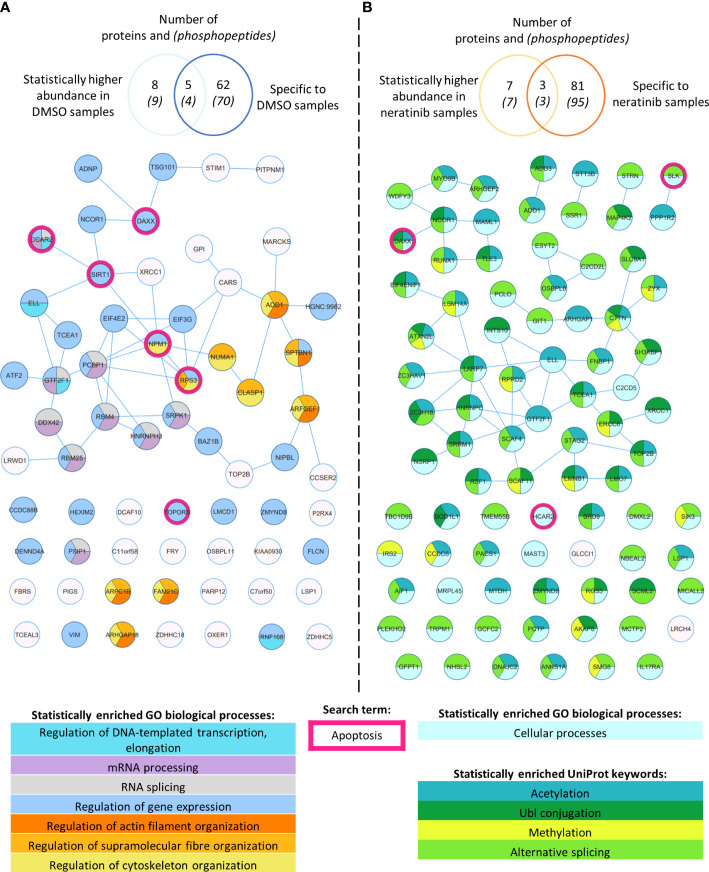
Phosphoproteomic analysis of human neutrophils shows that neratinib treatment induces changes in phosphorylated proteins regulating numerous biological processes. STRING identified interactions between phosphorylated proteins in the combined “DMSO-enriched” and “statistically higher abundance in DMSO” datasets **(A)** and the combined “neratinib-enriched” and “statistically higher abundance in neratinib” datasets **(B)**. Venn diagrams show the number of phosphopeptides and proteins they map to in each dataset and the overlapping proteins between the two datasets for each treatment group. STRING analysis of these datasets indicates interactions between proteins (lines). STRING also identified a number of Gene Ontology (GO) biological processes that were statistically enriched in both treatment groups, highlighted in color. The neratinib-enriched treatment group had only one statistically enriched biological process, and so statistically enriched keywords from the UniProt database are also shown. Both datasets were searched for the keyword “apoptosis,” and hit proteins were highlighted with a pink outline.

In order to define a larger set of phosphorylation sites putatively regulated by neratinib, we combined sites identified by statistical analysis with those that were enriched in either treatment group, i.e., phosphorylation sites identified in DMSO-treated samples but not neratinib samples, and vice versa. All phosphopeptides detected in three to four DMSO-treated samples and zero to one neratinib-treated sample were considered enriched with DMSO treatment, and all phosphopeptides detected in four to five neratinib- and zero to one DMSO-treated samples were considered enriched with neratinib treatment. This equated to 70 DMSO-enriched and 95 neratinib-enriched phosphorylated peptides, listed in **Supplementary File 1** (**Tables 2** and **3**, respectively), which mapped to 62 DMSO-enriched and 81 neratinib-enriched phosphorylated proteins. These two datasets, combined with the phosphopeptides that were identified as statistically more abundant in either treatment group, were analyzed using STRING (**Figure 2**).

STRING identified several functionally enriched biological processes, i.e., processes that were observed within these networks statistically more frequently than expected (**Figure 2**). In DMSO-treated samples, a number of enrichments were related to gene expression, e.g., mRNA processing and regulation of DNA-templated transcription and elongation, as well as regulation of actin filament, supramolecular fiber, and cytoskeleton organization (**Figure 2A**). These proteins might not be phosphorylated in neratinib-treated cells due to the onset of apoptosis and subsequent shutting down of these cellular processes. Within neratinib-treated samples, the only enriched biological process identified by STRING was “cellular processes” (**Figure 2B**). Several keywords extracted from the protein database UniProt were also identified as statistically enriched, including methylation, alternative splicing, and ubiquitin-like protein (Ubl) conjugation (**Figure 2B**). The latter is a key step in proteolysis, and these proteins are possibly phosphorylated in neratinib-treated cells due to the onset of apoptosis. In addition to analyzing the DMSO- and neratinib-treated samples separately, the datasets were combined and entered into STRING for analysis, as phosphorylation of one protein may result in the removal of a phosphate group from another (for example phosphatases that are regulated by phosphorylation). Biological processes enriched in this combined dataset were similar to those in the DMSO-treated samples, but also included regulation of protein polymerization and mRNA metabolic processes (**Supplementary Figure 3**). Analysis of this dataset using the online tool Reactome identified the top significantly enriched pathway being the Rho GTPase cycle (**Supplementary File 2**), which in neutrophils and other cell types regulate migration, phagocytosis, and efferocytosis by controlling cytoskeletal arrangements.

**Figure 3:**
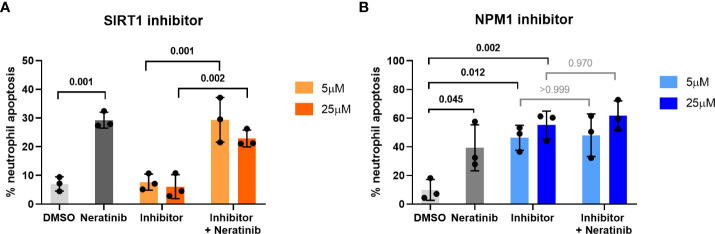
Pharmacological inhibition of candidates from the phosphoproteomic analysis reveals NPM1 as potentially being downstream of neratinib-induced neutrophil apoptosis. Human neutrophils were incubated with inhibitors of SIRT1 or NPM1, alone or in combination with 25 μM of neratinib, for 6 h, after which the levels of apoptosis were assessed by morphology. In both experiments, neratinib alone induced neutrophil apoptosis in comparison to the DMSO control, as expected. The inhibitor of SIRT1 (EX527) did not induce human neutrophil apoptosis **(A)**. The inhibitor of NPM1 (NSC348884) did induce human neutrophil apoptosis **(B)**, and no additional apoptosis was observed when this inhibitor was used in combination with neratinib. Each data point shows data from one healthy donor (*n* = 3); bars represent mean ± standard deviation. One-way ANOVA with multiple comparisons was used to calculate statistical significance; *p*-values were indicated.

Apoptosis was not identified as a statistically enriched process or keyword in any of the datasets analyzed; however, several proteins within both datasets have apoptosis listed as a UniProt keyword. These include NPM1, TOPORS, and SIRT1, which regulate p53 activity, and RBM25, which regulates the ratio of pro- and anti-apoptotic BCL2 isoforms (**Figure 2**, pink circles). These may be driving the pro-apoptotic mechanism of neratinib in human neutrophils. Two of these candidates, NPM1 and SIRT1, were tested to determine whether they induce apoptosis *via* the same mechanism as neratinib in human neutrophils, by assessing the ability of pharmacological inhibitors to induce neutrophil apoptosis, alone or in combination with neratinib. These were chosen due to their numerous interactions with other proteins within the STRING network, suggesting a possible pathway; RPS3 was also of interest for this reason, but an inhibitor could not be found. A pharmacological inhibitor of SIRT1 (EX527) did not induce neutrophil apoptosis (**Figure 3A**), which perhaps suggests that it is unlikely to be a candidate. However, inhibiting NPM1 (with the pharmacological inhibitor NSC348884) significantly increased neutrophil apoptosis (**Figure 3B**). The combination of the NPM1 inhibitor and neratinib did not result in an additional increase in apoptosis in comparison to the NPM1 inhibitor alone, suggesting an epistatic relationship.

### Neratinib treatment increases macrophage efferocytosis and reduces the number of neutrophil corpses in a murine model of LPS-induced acute lung injury

**Figure 4:**
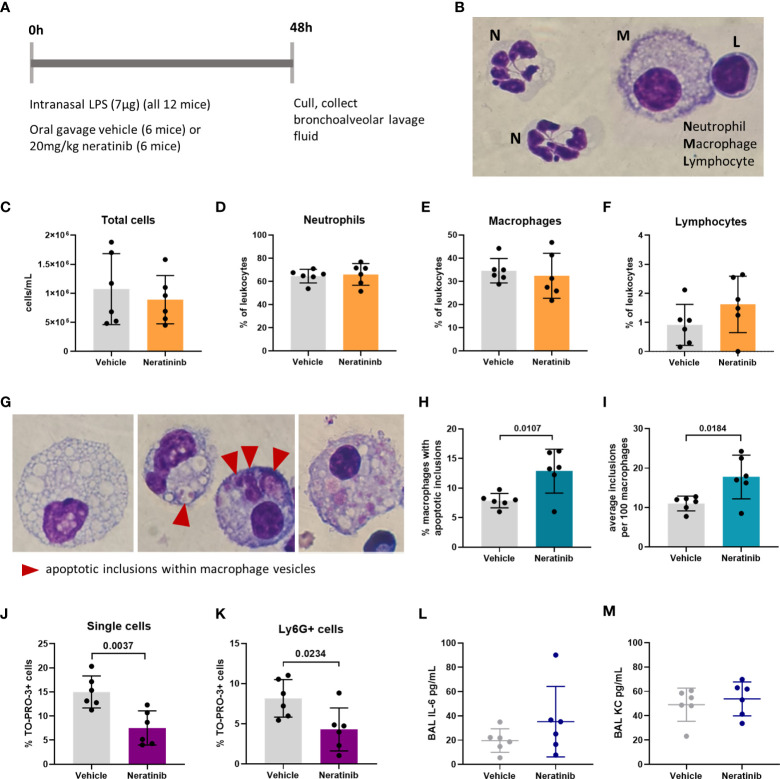
Neratinib treatment in a mouse LPS-induced acute lung injury model increases the rates of macrophage efferocytosis and reduces the number of neutrophil corpses in bronchoalveolar lavage fluid. Schematic of the treatment protocol **(A)**. Cytospins of BAL were stained with Kwik-Diff and examined by light microscopy, and neutrophils, macrophages, and lymphocytes were identified by morphology **(B)**. Total cells in BAL were counted using the hemocytometer counting chamber **(C)**, and the percentage of neutrophils **(D)**, macrophages **(E)**, and lymphocytes **(F)** in BAL were unchanged between treatment groups. The engulfment of cell debris by macrophages is visible as inclusions within intracellular vesicles, which can be identified with Kwik-Diff staining and light microscopy **(G**, red arrows**)**. Many macrophages contained vesicles, but these were empty of debris **(G**, left panel**)**. Cell debris may be broken into small pieces within a vesicle **(G**, middle panel, left**)** or fill the vesicles **(G**, middle panel, right**)**. Macrophages may contain one or more vesicles with inclusions; in some cases, too many to count accurately **(G**, right panel**)**; if above 6, the number of inclusions was recorded as 6. Both the percentage of macrophages containing any inclusions **(H)** and the total number of inclusions per 100 macrophages **(I)** were increased in the neratinib treatment group. Cells in BAL were also analyzed by flow cytometry, and the percentage of TO-PRO-3+ cells **(J)** and TO-PRO-3+ neutrophils (Ly6G+ cells) **(K)** was reduced in neratinib-treated mice. Concentrations of the cytokines IL-6 **(L)** and KC **(M)** were analyzed in BAL supernatant by ELISA and were unchanged between treatment groups. Each data point in the graphs represents data from one mouse (*n* = 6 per treatment group); bars show mean ± standard deviation. Unpaired *t*-tests were used for statistical analysis; *p*-values were indicated, where *p* <0.05.

To determine whether neratinib may ultimately be beneficial as a treatment for patients with chronic inflammatory diseases such as COPD, we used several murine lung injury models. Our previous research showed that the tyrphostin AG825 increased neutrophil apoptosis and efferocytosis by macrophages in an acute LPS-induced lung injury model. We used the same model to treat mice with either neratinib (20 mg/kg) or vehicle (**Figure 4A**). After culling all mice 48 h post-treatment, cells in BAL fluid were analyzed and identified as neutrophils, macrophages, or lymphocytes based on morphology (**Figure 4B**). The total cell number in BAL from each mouse was unchanged with neratinib treatment (**Figure 4C**), as was the percentage of neutrophils, macrophages, and lymphocytes (**Figures 4D–F**). Macrophage efferocytosis was quantified by identifying inclusions of dead cells or cell debris within macrophage vesicles (**Figure 4G**). The majority of macrophages contained no apoptotic inclusions and were not considered to be undergoing efferocytosis (**Figure 4G**, left panel). As other macrophages had multiple vesicles containing inclusions, the total number of inclusions per 100 macrophages was calculated, as well as the percentage of macrophages containing any inclusions; both measures of efferocytosis were significantly increased with neratinib treatment (**Figures 4H**, **I**).

As we observed an increase in efferocytosis, we examined whether the percentage of free dead cells in BAL was altered with neratinib treatment. Cells were analyzed by flow cytometry, staining for Ly6G to identify neutrophils, Annexin V to identify apoptotic cells, and the viability dye TO-PRO-3 (**Supplementary Figure 4A**). Since TO-PRO-3 can only enter cells that have lost membrane integrity (dead cells), these are referred to as cell corpses. The majority of TO-PRO-3+ cells also stained for Annexin V (**Supplementary Figures 4B, C**), suggesting that these cells either underwent apoptosis and subsequent secondary necrosis or have directly lost membrane integrity. The percentage of both TO-PRO-3+ cells and TO-PRO-3+ neutrophils was significantly decreased in the neratinib treatment group, supporting a role for neratinib-enhanced efferocytosis in preventing the accumulation of cell corpses, particularly neutrophil corpses (**Figures 4J, K**). Neratinib treatment did not alter the percentage of viable or apoptotic cells or neutrophils (**Supplementary Figures 4D–G**).

We also analyzed the concentration of cytokines in the supernatant from BAL by ELISA to determine if neratinib might be reducing inflammatory cytokine levels. Both IL-6 and KC (also known as CXCL1 or GROα) were unchanged between the neratinib and control treatment groups (**Figures 4L, M**).

An increase in the rate of efferocytosis, and a decrease in the number of cell corpses, particularly neutrophil corpses, could be beneficial in the resolution of inflammation. We followed up these promising results in a chronic murine model of lung disease to determine if neratinib is beneficial in a chronic inflammatory environment.

### Neratinib treatment reduces inflammatory cytokines in a chronic murine lung injury model

**Figure 5:**
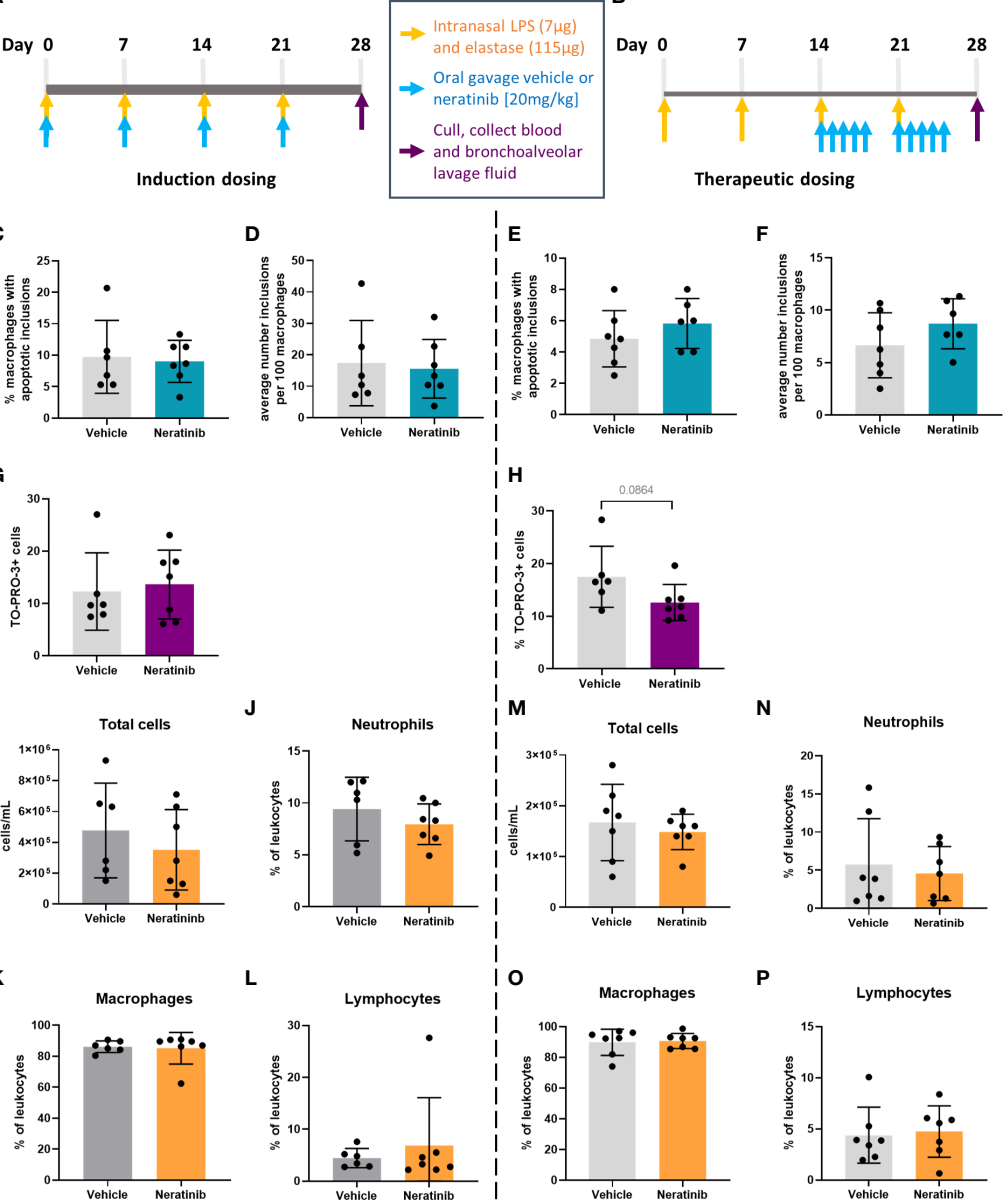
No changes in efferocytosis or cell corpse numbers were observed with neratinib treatment in chronic models of lung disease. Two different dosing protocols were used for neratinib treatment in the chronic lung disease model: induction dosing **(A)** and therapeutic dosing **(B)**. Uptake of cell debris by macrophages was unchanged between treatment groups in both the induction dosing **(C, D)** and therapeutic dosing **(E, F)** models. The percentage of TO-PRO-3+ cells in BAL, assessed by flow cytometry, was unchanged in the induction dosing model **(G)** and the therapeutic dosing model **(H)**. Leukocyte number in BAL and the percentage of neutrophils, macrophages, and lymphocytes were similarly unchanged across both models **(I–P)**. Each data point in graphs represents data from one mouse; bars show mean ± standard deviation. Induction dosing study: *n* = 6 vehicle-treated mice, *n* = 7 neratinib-treated mice; therapeutic dosing study: *n* = 7 mice in each treatment group. Unpaired *t*-tests were used for statistical analysis.

To determine if neratinib may have therapeutic benefit in a chronic model of lung inflammation, mice were given weekly intranasal doses of LPS and elastase over 4 weeks, which is known to result in COPD-like features within 4 weeks. Elastase induces widespread tissue damage and is a profound inducer of emphysema-like damage and bronchitis. LPS is an excellent inflammatory stimulus and also models the contribution of infection (compared to isolated cigarette smoke models for example). Overall, these treatments induce airway inflammation, emphysematous changes, and functional impairment comparable to COPD. We tested two different dosing schedules for neratinib administration: one in which neratinib was given at the same time as the LPS/elastase (induction dosing) (**Figure 5A**) and the second in which neratinib treatment began 2 weeks after disease onset (therapeutic dosing) (**Figure 5B**). In both cases, mice were culled on day 28, and BAL and blood were collected for analysis. All mice developed lung inflammation, with evidence of epithelial damage and areas of alveolar enlargement, as determined by histological analysis.

In contrast with the acute lung injury study, efferocytosis by macrophages was not significantly modified by neratinib treatment (**Figures 5C–F**). Cells within BAL were also analyzed by flow cytometry, and neither the neratinib treatment schedule altered the percentage of TO-PRO-3+ cells, although a trend for decreased cell corpses was observed in the therapeutic model (**Figures 5G, H**). The total number of leukocytes in BAL samples and the percentage of neutrophils, macrophages, and lymphocytes were similarly unchanged between the treatment groups in both studies (**Figures 5I–P**).

**Figure 6:**
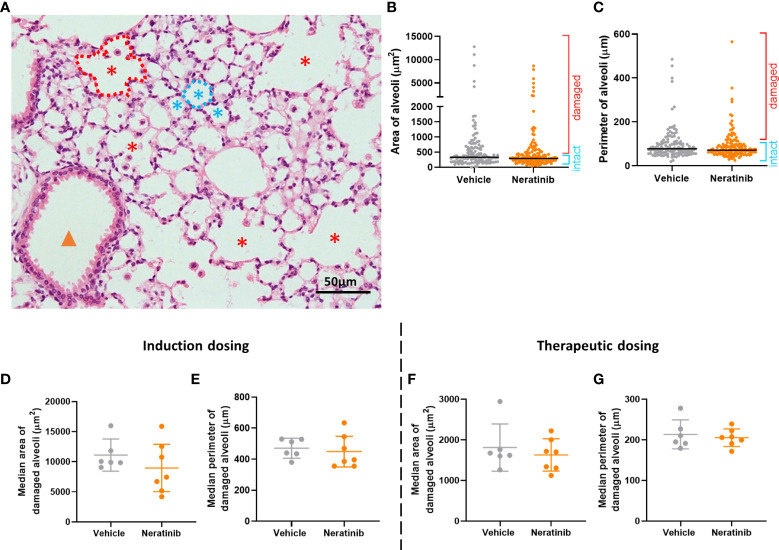
Neratinib does not alter alveolar area or perimeter in chronic mouse models of lung disease. Fixed mouse lung tissue slices were stained with H&E **(A)**. Alveoli were identified by a lumen surrounded by characteristically thin squamous epithelial cells and classified as intact (blue stars) or damaged (red stars) and differentiated from bronchioles (orange arrowhead) as described in the *Materials and methods*. Area and perimeter measurements were calculated using the outline tool (dotted lines) in NIS-Elements. Initially, random alveoli were selected for analysis; however, this resulted in the majority measured being intact **(B, C)**. Each dot represents one alveolus, line at median; 20 alveoli measured across two lung slices per mouse, in the induction dosing study. To assess only damaged alveoli, a lower threshold of 500 μm^2^ for area and 75 μm for perimeter was set, and alveoli below these sizes were excluded from the analysis. Median damaged alveolar area and perimeter per mouse were assessed, and no differences between treatment groups were found with either the induction dosing protocol **(D, E)** or the therapeutic dosing protocol **(F, G)**. **(D–G)** Each dot represents one median alveolar area/perimeter per mouse, calculated from 20 alveolar measurements across two lung slices. Bars show mean ± standard deviation. Unpaired *t*-test was used to compare treatment groups.

**Figure 7:**
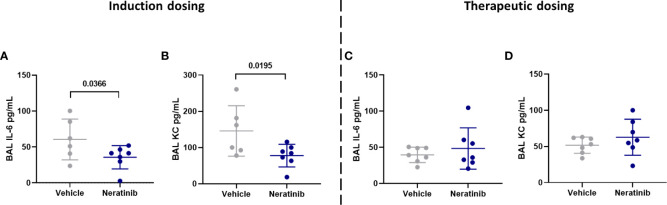
Neratinib treatment reduces the levels of cytokines IL-6 and KC in BAL in the induction dosing model of lung disease. Cytokines were measured in BAL supernatant by ELISA. Interleukin-6 (IL-6) and KC were reduced in neratinib-treated mice in the induction dosing model **(A, B)**, whereas these were unchanged in the therapeutic dosing model **(C, D)**. Each data point represents data from one mouse; bars show mean ± standard deviation. Unpaired *t*-tests were used for statistical analysis; *p*-values were indicated.

To determine whether neratinib treatment altered the damage to the lung tissue observed in this model, histological analysis was carried out on lung tissue slices (**Figure 6A**). Both the area and the perimeter of alveoli were measured. When alveoli were selected at random for analysis, the majority selected were intact (**Figures 6B, C**). In order to determine the effect of neratinib on the size of damaged alveoli, the parameters of selection were altered so that only larger alveoli (>500 μm^2^ for area and >75 μm for perimeter) were analyzed. The median damaged alveolar area and perimeter per mouse were not significantly different following neratinib treatment, in neither the induction dosing nor the therapeutic neratinib dosing protocols (**Figures 6D–G**). The concentration of inflammatory cytokines IL-6 and KC was analyzed in the BAL supernatant. Unlike the acute lung injury study, both IL-6 and KC were significantly reduced with the induction dosing method (**Figures 7A, B**); however, they were unchanged with therapeutic dosing of neratinib (**Figures 7C, D**).

**Figure 8:**
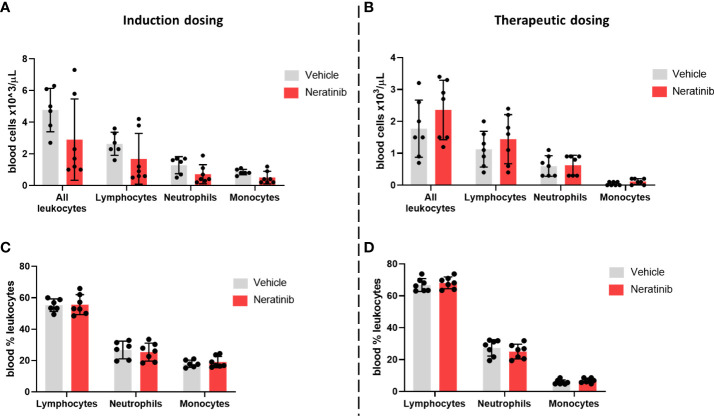
Neratinib does not alter the numbers or distribution of circulating leukocytes in chronic mouse models of lung disease. Leukocytes in mouse whole-blood samples were analyzed using an automated hematology blood analyzer, which counts total leukocytes as well as differentiates between lymphocytes, neutrophils, and monocytes; the percentages of each leukocyte subset are also analyzed. No differences in the number of leukocytes, or their subsets, were identified between the vehicle- and neratinib-treated groups in the induction dosing **(A)** or therapeutic dosing **(B)** model, and similarly, no differences in the percentages of lymphocytes, neutrophils, and monocytes were found between treatment groups in the induction **(C)** or therapeutic **(D)** dosing model. Each data point represents data from one mouse; bars show mean ± standard deviation. Unpaired *t*-tests were used to compare treatment groups within each cell type; *p*-values were indicated where appropriate.

Blood samples were analyzed using an automated hematology analyzer to enumerate circulating leukocyte numbers and to differentiate between neutrophils, monocytes, and lymphocytes. We found no changes either in the number of circulating leukocytes (**Figures 8A, B**) or in the percentage of neutrophils, monocytes, and lymphocytes (**Figures 8C, D**) between the vehicle and neratinib treatment groups, with either dosing protocol.

## Discussion

The suppression of neutrophil apoptosis and the release of neutrophilic inflammatory mediators in injured tissue are essential for efficient pathogen clearance, and individuals with defective neutrophils or chronic neutropenia experience life-threatening infections (20, 21). However, in chronic inflammatory diseases such as COPD, the continual release of neutrophilic reactive oxygen species, elastase, and other proteolytic enzymes destroys alveolar epithelial cells, drives emphysematous changes, and damages the alveolar attachments that support bronchioles, resulting in their premature collapse (22, 23). Modifying neutrophils in this disease therefore may have therapeutic potential, although currently there are no therapeutics that do this. A number of studies have shown the beneficial effects of inducing apoptosis and reducing the functional activity of neutrophils, as well as increasing the rate of efferocytosis by macrophages, in various experimental inflammatory models (24–26). Here, we show that neratinib, an ErbB inhibitor used to treat breast cancer, may be able to reduce acute inflammation in the lungs, by reducing the number of neutrophil corpses and increasing the rates of efferocytosis.

The rapid removal of apoptotic neutrophils from an inflammatory environment before they become necrotic is crucial to prevent the highly histotoxic intracellular contents from inducing tissue damage and further inflammation (27). The process of efferocytosis itself is also a key step in inflammation resolution, skewing macrophages toward an anti-inflammatory phenotype in which they release IL-10, TGFβ, and prostaglandin E2 to facilitate wound healing, and suppress the production of pro-inflammatory cytokines such as IL-1β, CXCL-8, TNFα, and GM-CSF (28–30). Interestingly, research using cell therapy (injected stem cells) to ameliorate inflammatory disease in mouse models has suggested that apoptosis of the injected cells, and the subsequent efferocytosis by macrophages, is causing the immunosuppressive effects, rather than bioactivity of the stem cells themselves (31). In mouse models of pneumococcal pneumonia, instilled apoptotic macrophages are engulfed by resident tissue macrophages, which resulted in decreased neutrophilic inflammation and inflammatory cytokine levels in the lungs and a lower risk of bacteremia (32).

It is unclear in our studies whether neratinib is directly regulating macrophage efferocytosis or upregulating apoptosis and thereby increasing the amount of material available to engulf. Establishing the identity of the macrophage vesicle contents would be helpful to investigate this, for example by biochemical co-staining for markers of neutrophils and apoptosis. This is particularly important since other studies show that alveolar macrophages ingest apoptotic alveolar epithelial cells as well as apoptotic neutrophils in the lungs and airways (33, 34). There is very little literature suggesting that ErbB signaling has a role in efferocytosis, although a recent study in *Drosophila* from our group shows that overexpression of the cardinal EGF ligand, Spitz, impairs macrophage-mediated apoptotic cell clearance (35). While this supports our current findings, there are likely to be a number of mechanisms at play, including EGF acting as a chemoattractant to “distract” the macrophages from apoptotic cell clearance, or EGFR signaling from other tissues polarizing the macrophages toward a phenotype less efficient at efferocytosis (35).

The binding of macrophage receptors to apoptotic markers such as phosphatidylserine activates intracellular macrophage signaling pathways including PI3K-AKT, STAT-SOCS, and Rho GTPases, and suppresses NFκB and IFNα/β signaling, resulting in the engulfment of the apoptotic cell (36). Our phosphoproteomic analysis using Reactome identified an enrichment in the Rho GTPase cycle, a known controller of efferocytosis and phagocytosis (37, 38).

Rho GTPases are regulated by GTPase-activating proteins (GAPs) and guanine nucleotide exchange factors (GEFs) (39). Our phosphoproteomic analysis of human neutrophils identified three of these phosphorylated proteins in the neratinib-enriched dataset (ARHGAP1, ARHGEF2, and MYO9B) and one in the DMSO-enriched dataset (ARHGAP18). RhoA, which inhibits the Rho GTPase cycle and thus efferocytosis, is itself inhibited by the cholesterol-lowering drugs statins (40). Treatment with lovastatin upregulated efferocytosis in mouse lungs *in vivo* and also in human macrophages from patients with COPD *in vitro* (41). As we carried out the phosphoproteomic experiments with human neutrophils, it is not possible to tell if neratinib is regulating these pathways in macrophages in our acute lung injury mouse model. Further work, such as targeted immunostaining of macrophages for candidate molecules identified in this current study, would be required to draw such conclusions.

Rho GTPases regulate other neutrophil functions including adhesion, chemotaxis, and recruitment by controlling cytoskeletal arrangements (37, 42). Our phosphoproteomic analysis indicated that neratinib regulates actin filament assembly and cytoskeletal organization, which also control migration. In the zebrafish larvae, neratinib treatment resulted in a reduction in neutrophil numbers at the tail fin injury site, suggesting an impairment in neutrophil migration. ErbBs are known to regulate migration, with research demonstrating that overamplification of ErbB receptors on tumor cells can induce epithelial–mesenchymal transition (leading to metastasis), migration, and tumor invasion (43). Neratinib specifically reduces the migration of gastric adenocarcinoma cells *in vitro* (44). Our data suggest that neratinib-induced inhibition of migration might be due to the regulation of Rho GTPase pathways, although again further validation is required to conclude this.

The mechanism by which neratinib is promoting apoptosis of neutrophils *in vitro* is still unclear, although the phosphoproteomic analysis has elucidated several potential candidates. Nucleophosmin 1 (NPM1) is a protein with a range of intracellular functions including ribosome biogenesis, protein chaperoning, histone assembly, and regulation of the tumor suppressor p53 and is often mutated or overexpressed in cancer cells, contributing to carcinogenesis (45). Our data show that a pharmacological inhibitor of NPM1 induces neutrophil apoptosis, both alone and in the presence of neratinib, suggesting that these two inhibitors may be inducing apoptosis in neutrophils *via* the same mechanism. We detected phosphorylation of NPM1 at serine 4 and serine 10 in three out of four DMSO-treated samples and in none out of five neratinib-treated samples, suggesting a potential role in the induction of apoptosis with neratinib treatment. Other sources have linked these specific phosphorylation events to cell death; for example, irradiated basal epithelial cells show dephosphorylation of NPM1 at serine 4 (46). Inactivation of the serine 10 and serine 70 phosphorylation sites of NPM1 induces cell cycle arrest in mutant lymphoblastoid cells and also positively regulates the activity of cyclin-dependent kinase 1, a key regulator of the cell cycle (47).

Targeting EGFR signaling as a therapeutic strategy for inflammatory lung diseases has been investigated by others, although not in the context of modifying neutrophils specifically. Bronchial epithelial cells from patients with COPD have increased production of CXCL8 and increased phosphorylation of EGFR and AKT; however, all were reduced *in vitro* with erlotinib, a clinical EGFR inhibitor (48). The binding of the EGFR ligand amphiregulin was shown to be essential for TGFβ-dependent pulmonary fibrosis in mouse models (49). The EGFR signaling pathway was also shown to contribute to the loss of muscle function that many patients with COPD experience (50). Rhinovirus infection upregulates mucin production in the airways *via* the NFκB and EGFR pathways, and this was suppressed in mouse models of COPD exacerbations using an EGFR inhibitor (51, 52). A clinical trial of an inhaled EGFR antagonist, however, did not reduce mucus production in patients with COPD (53). Although EGFR inhibitors have to date only been approved for cancer treatment, other research does suggest that they have potential benefit for inflammatory lung diseases such as COPD.

The results from our chronic mouse models of lung inflammation suggest that neratinib (as dosed here) is less effective over longer time periods. The reduction of cytokines KC and IL-6 in the induction dosing model of neratinib treatment is promising, although it is unclear if this parameter alone would confer therapeutic benefit. The reduction in KC is particularly interesting as this cytokine is a key recruiter of neutrophils to sites of inflammation or infection (54). Other murine acute lung injury models show that depletion of KC correlates with decreased early neutrophil recruitment and improved histopathology scores, suggesting a potential therapeutic benefit (55). Blood biomarkers of cytokines such as IL-6 have also been shown to correlate with disease severity in patients with COPD (56). We measured no change in the number or proportion of neutrophils in BAL, and it may be that other inducers of neutrophil recruitment in mice such as LTB4 and CXCL5 (57, 58) render the abrogation of KC redundant. We also measured no changes in cytokine levels with the therapeutic dosing protocol of neratinib or in the acute lung injury model. This suggests that treatment is required earlier in the course of the disease to show efficacy and requires either more than one dose, or longer than 48 h, for the reduction to be observed.

Since neratinib showed the most efficacy in the acute lung inflammation mouse model, for COPD patients, it may be that this drug would most benefit those experiencing exacerbations, rather than as a long-term treatment option. Further pre-clinical models and clinical trials would ultimately be required to determine any potential benefit; however, this work has given additional evidence to support neratinib repurposing, narrowed down the inflammatory context in which it may have therapeutic potential, and further defined the intracellular regulators by which neratinib exerts its anti-inflammatory effects.

## Data availability statement

The mass spectrometry proteomics data has been deposited in the ProteomeXchange Consortium via the PRIDE partner repository, with the dataset identifier PXD035125.

## Ethics statement

The studies involving human participants were reviewed and approved by South Sheffield Research Ethics Committee reference number STH13927. The patients/participants provided their written informed consent to participate in this study. The animal study was reviewed and approved by Animal Welfare and Ethical Review Body at the University of Sheffield.

## Author contributions

KH, CW, SM, and MC performed the experiments. KH, CW, SM, MC, HM, KB, LP, and SR contributed to the experimental design and data analysis. LP, SR, and HM provided home office or ethical permission to study the animals/human samples. LP and SR secured the funding. KH, LP, and SR wrote the manuscript. All authors reviewed and edited the drafts of the manuscript. All authors contributed to the article and approved the submitted version.

## Funding

This work was supported by a Medical Research Council (MRC) Programme Grant to SR (MR/M004864/1), a Rosetrees Trust grant to LP (100179), and the Faculty of Medicine, Dentistry and Health Doctoral Academy Scholarship to KH.

## Acknowledgments

We thank the Biological Services Aquarium staff and the Biological Services Unit staff for their assistance with zebrafish and mouse husbandry. We would also like to thank the following staff in facilities at the University of Sheffield: Fiona Wright in the IICD Histology Hub, the Wolfson Light Microscopy Facility, the Medical School Flow Cytometry Core Facility, and the biOMICS Mass Spectrometry Facility. Lastly, we would like to thank the volunteers who donated blood for this research. This manuscript is available on the pre-print server, BioRxiv (59). Work in this paper is also published in the thesis of KH, available on the White Rose eThesis Online Repository (60).

## Conflict of interest

The authors declare that the research was conducted in the absence of any commercial or financial relationships that could be construed as a potential conflict of interest.

## Publisher’s note

All claims expressed in this article are solely those of the authors and do not necessarily represent those of their affiliated organizations, or those of the publisher, the editors and the reviewers. Any product that may be evaluated in this article, or claim that may be made by its manufacturer, is not guaranteed or endorsed by the publisher.

## Supplementary material

Supplementary File 1 Phosphoproteomics dataset.

Supplementary File 2 Reactome analysis of phosphorylated proteins enriched and statistically regulated in both treatment groups.

Supplementary Figure 1 Validation of the dataset by identifying phosphorylated proteins downstream of cAMP signalling pathways, and downstream of ErbB signaling pathways. Phosphorylated proteins downstream of cAMP signaling pathways were identified from the dataset **(A)**. cAMP binds to the regulatory subunit of inactive protein kinase A (PKA), resulting in the dissociation and activation of catalytic PKA subunits, which in turn phosphorylate a number of downstream targets, such as BRAF, GSK3A and MAPK proteins, and transcription factors AFT and CREB in the nucleus. cAMP is also converted to 5’ AMP by PDE enzymes. The data was analyzed by noting the number of samples in which a phosphorylated peptide mapping to a protein was detected in the DMSO and neratinib treatment groups. This is represented as a ratio of the total number of samples in each treatment group. For example, a phosphorylated peptide mapping to CREB1 was detected in 3/4 (0.75) DMSO treated samples and 5/5 (1) neratinib treated samples. In some cases, multiple phosphorylated peptides mapping to the same protein were identified. The same approach was taken to identify phosphorylated peptides mapping to proteins downstream of ErbB signalling pathways **(B)**.

Supplementary Figure 2 Statistical analysis of phosphopeptide abundance between treatment groups. Statistical analysis of the phosphoproteomics dataset identified 16 phosphopeptides with significantly different abundances between the neratinib and DMSO treated neutrophils, at 5% permutation-based false discover rate (FDR) using a paired student’s t test. This dataset was input into STRING, and one interaction between two phosphorylated proteins increased with neratinib treatment, MAML1 and NCOR1, was identified **(B)**.

Supplementary Figure 3 STRING analysis of phosphorylated proteins enriched and statistically regulated in both treatment groups. The DMSO- and neratinib-enriched phosphorylated proteins, and the statistically regulated phosphorylated proteins, were combined into one dataset and analysed in STRING. A selection of the biological processes that were identified as statistically enriched are highlighted in colour. Although not statistically enriched, a number of proteins are also involved in the regulation of apoptosis (pink outlines). Lines indicate interactions between proteins; some proteins have multiple interactions, whereas others have none.

Supplementary Figure 4 Flow cytometry analysis of BAL cells from the LPS-induced acute lung injury mouse model. Cells in BAL were analyzed by flow cytometry. Single cells were initially gated **(A**, left panel**)**, followed by Ly6G+ cells to identify neutrophils **(A**, middle panel**)**. Annexin-V and TO-PRO-3 staining were then used to categorize cells as viable (negative for Annexin-V and TO-PRO-3), apoptotic (Annexin-V+), dead (TO-PRO-3+) and secondary necrotic (Annexin-V+ and TO-PRO-3+) **(A**, right panel**)**. The single cells **(B)** and neutrophils **(C)** from each mouse BAL sample were categorized as such. Each bar represents data from one mouse. The percentage of viable cells **(D)** and viable neutrophils **(E)** was unchanged between treatment groups, as was the percentage of apoptotic cells **(F)** and apoptotic neutrophils **(G)**. **(D–G)**: each data point represents data from one mouse; bars show mean ± standard deviation. Unpaired t tests used for statistical analysis; no significant differences found between vehicle and neratinib treatment groups.
