# Supplementary material for: The EGFR/ErbB inhibitor neratinib modifies the neutrophil phosphoproteome and promotes apoptosis and clearance by airway macrophages
Source: Front Immunol. 2022 Jul 28;13:956991. doi: 10.3389/fimmu.2022.956991 (PMC9371615; doi:10.3389/fimmu.2022.956991)
Supplement: Supplementary File 2 — Reactome analysis of phosphorylated proteins enriched and statistically regulated in both treatment groups. [file DataSheet_1.pdf]

4. Most significant pathways

The following table shows the 25 most relevant pathways sorted by p-value.

Pathway name	Entities				Reactions	
	found	ratio	p-value	FDR*	found	ratio
RHO GTPase cycle	15 / 460	0.031	6.70e-05	0.02	33 / 91	0.007
Alternative Lengthening of Telomeres (ALT)	2 / 2	1.33e-04	1.97e-04	0.02	2 / 2	1.47e-04
Defective Inhibition of DNA Recombination at Telomere	2 / 2	1.33e-04	1.97e-04	0.02	2 / 2	1.47e-04
Diseases of Telomere Maintenance	2 / 2	1.33e-04	1.97e-04	0.02	2 / 2	1.47e-04
Defective Inhibition of DNA Recombination at Telomere Due to ATRX Mutations	2 / 2	1.33e-04	1.97e-04	0.02	1 / 1	7.33e-05
Defective Inhibition of DNA Recombination at Telomere Due to DAXX Mutations	2 / 2	1.33e-04	1.97e-04	0.02	1 / 1	7.33e-05
Signaling by Rho GTPases	18 / 709	0.047	2.86e-04	0.025	41 / 203	0.015
Signaling by Rho GTPases, Miro GTPases and RHOBTB3	18 / 725	0.048	3.73e-04	0.028	41 / 212	0.016
XBP1(S) activates chaperone genes	6 / 95	0.006	4.29e-04	0.028	3 / 47	0.003
IRE1alpha activates chaperones	6 / 101	0.007	5.89e-04	0.035	3 / 53	0.004
RUNX3 regulates RUNX1-mediated transcription	2 / 4	2.65e-04	7.77e-04	0.042	2 / 2	1.47e-04
RHOF GTPase cycle	4 / 46	0.003	0.001	0.063	2 / 3	2.20e-04
RHOBTB1 GTPase cycle	3 / 24	0.002	0.002	0.088	1 / 2	1.47e-04
SUMO E3 ligases SUMOylate target proteins	7 / 183	0.012	0.003	0.109	10 / 131	0.01
RNA polymerase II transcribes snRNA genes	5 / 94	0.006	0.003	0.109	9 / 11	8.06e-04
SUMOylation	7 / 192	0.013	0.003	0.127	10 / 140	0.01
Signaling by ALK fusions and activated point mutants	4 / 66	0.004	0.005	0.143	24 / 29	0.002
Signaling by ALK in cancer	4 / 66	0.004	0.005	0.143	25 / 37	0.003
Cohesin Loading onto Chromatin	2 / 10	6.64e-04	0.005	0.143	2 / 2	1.47e-04
Unfolded Protein Response (UPR)	6 / 155	0.01	0.005	0.143	3 / 94	0.007
Tat-mediated HIV elongation arrest and recovery	3 / 36	0.002	0.006	0.143	3 / 3	2.20e-04
Pausing and recovery of Tat-mediated HIV elongation	3 / 36	0.002	0.006	0.143	2 / 2	1.47e-04
RHOBTB GTPase Cycle	3 / 36	0.002	0.006	0.143	2 / 4	2.93e-04
HIV elongation arrest and recovery	3 / 37	0.002	0.006	0.143	3 / 3	2.20e-04

Pathway name	Entities				Reactions	
	found	ratio	p-value	FDR*	found	ratio
**Pausing and recovery of HIV elongation**	3 / 37	0.002	0.006	0.143	2 / 2	1.47e-04

* False Discovery Rate
