# Supplementary figures and images for: The EGFR/ErbB inhibitor neratinib modifies the neutrophil phosphoproteome and promotes apoptosis and clearance by airway macrophages
Source: Front Immunol. 2022 Jul 28;13:956991. doi: 10.3389/fimmu.2022.956991 (PMC9371615; doi:10.3389/fimmu.2022.956991)

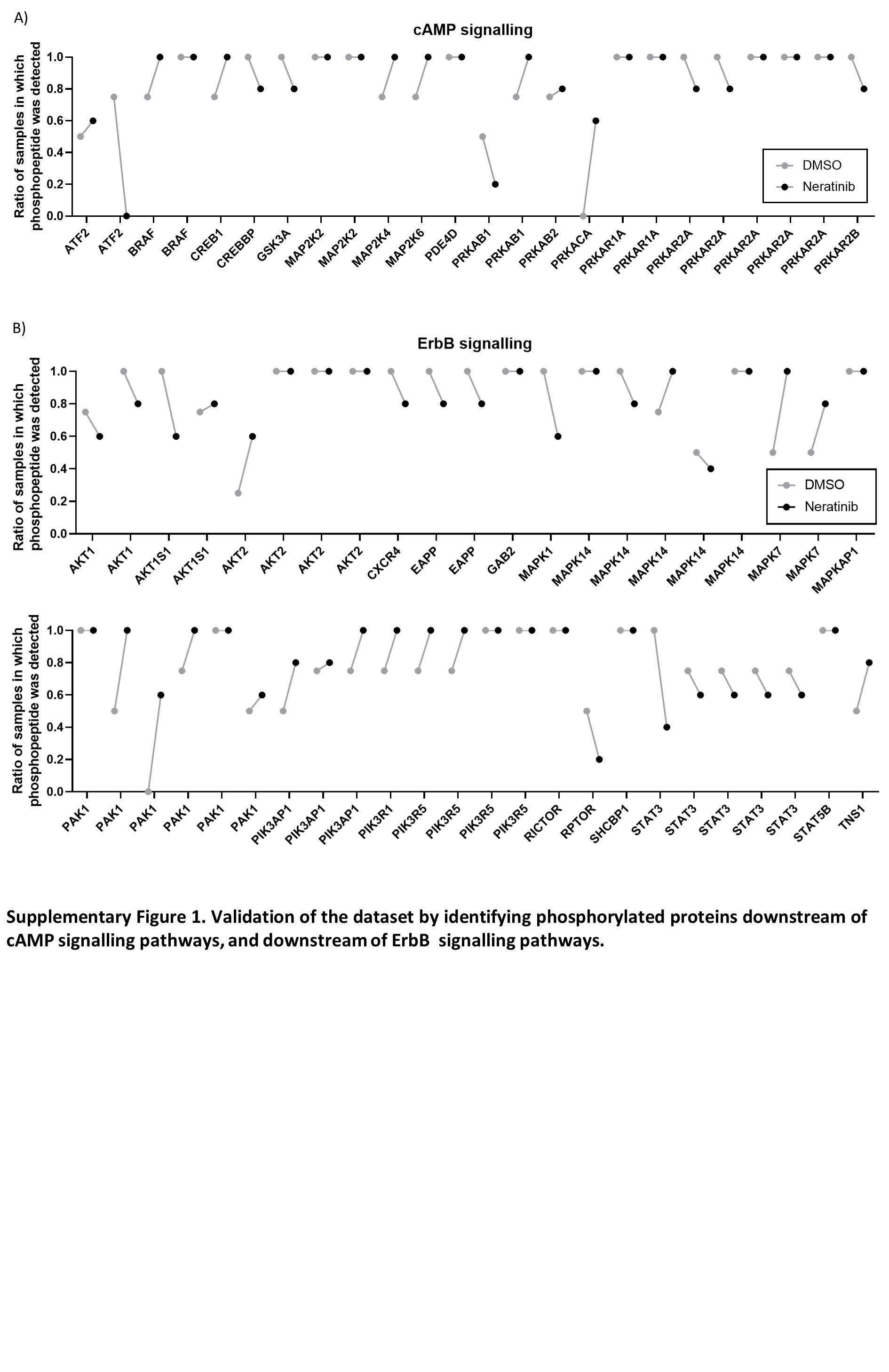

Supplement: Supplementary Figure 1 — Validation of the dataset by identifying phosphorylated proteins downstream of cAMP signalling pathways, and downstream of ErbB signaling pathways. Phosphorylated proteins downstream of cAMP signaling pathways were identified from the dataset (A). cAMP binds to the regulatory subunit of inactive protein kinase A (PKA), resulting in the dissociation and activation of catalytic PKA subunits, which in turn phosphorylate a number of downstream targets, such as BRAF, GSK3A and MAPK proteins, and transcription factors AFT and CREB in the nucleus. cAMP is also converted to 5’ AMP by PDE enzymes. The data was analyzed by noting the number of samples in which a phosphorylated peptide mapping to a protein was detected in the DMSO and neratinib treatment groups. This is represented as a ratio of the total number of samples in each treatment group. For example, a phosphorylated peptide mapping to CREB1 was detected in 3/4 (0.75) DMSO treated samples and 5/5 (1) neratinib treated samples. In some cases, multiple phosphorylated peptides mapping to the same protein were identified. The same approach was taken to identify phosphorylated peptides mapping to proteins downstream of ErbB signalling pathways (B). [file Image_1.tif]

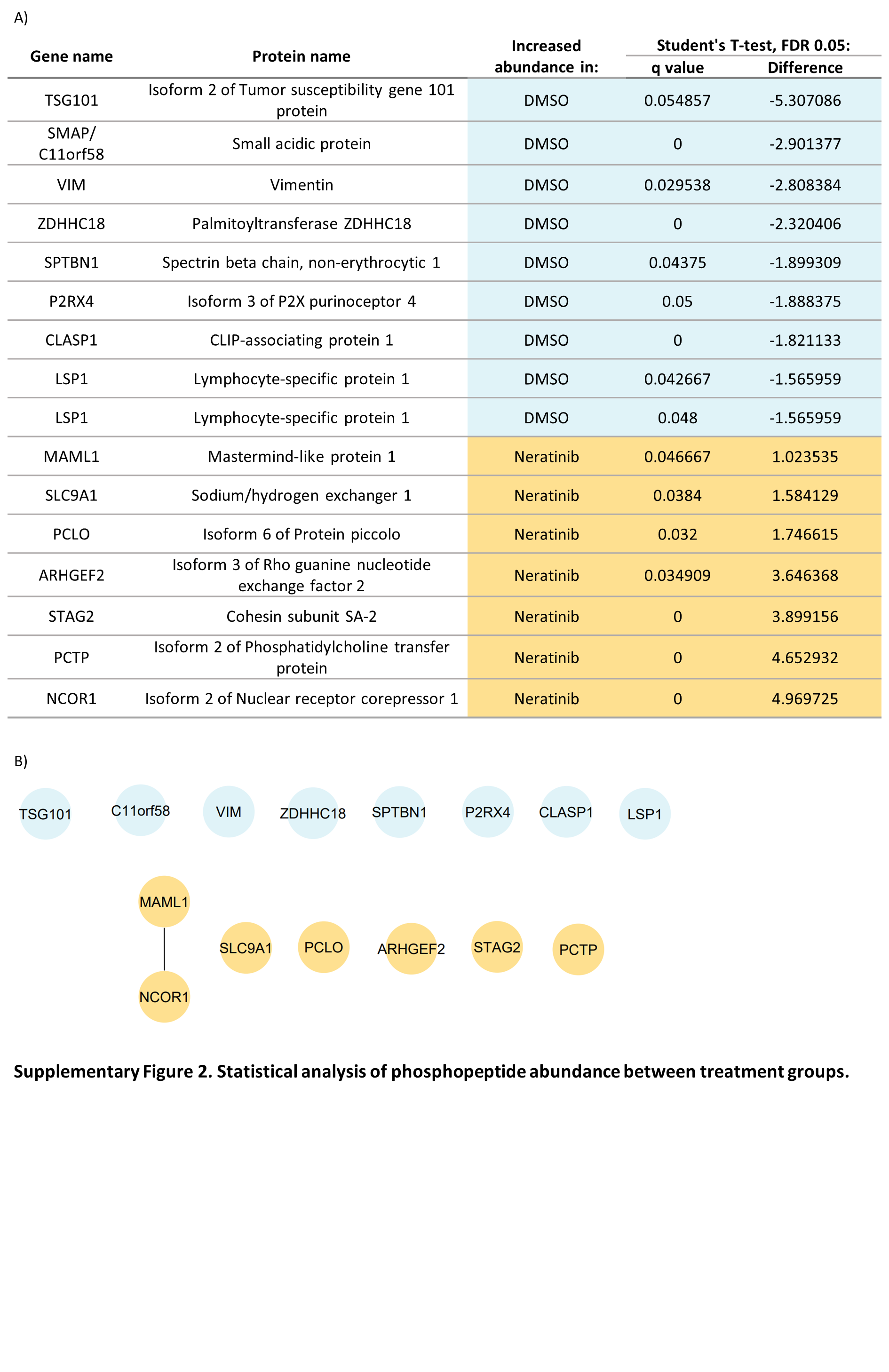

Supplement: Supplementary Figure 2 — Statistical analysis of phosphopeptide abundance between treatment groups. Statistical analysis of the phosphoproteomics dataset identified 16 phosphopeptides with significantly different abundances between the neratinib and DMSO treated neutrophils, at 5% permutation-based false discover rate (FDR) using a paired student’s t test. This dataset was input into STRING, and one interaction between two phosphorylated proteins increased with neratinib treatment, MAML1 and NCOR1, was identified (B). [file Image_2.tif]

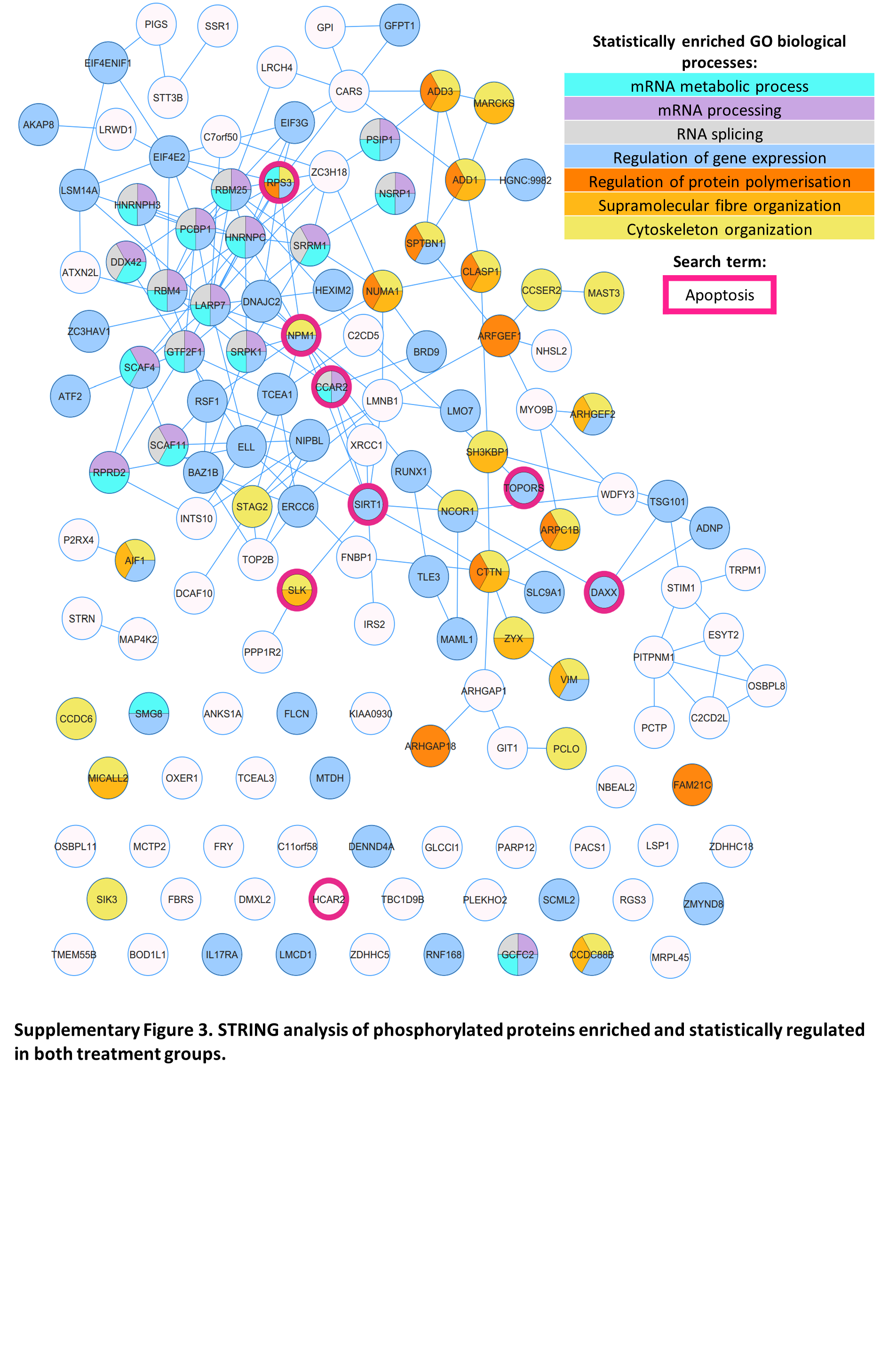

Supplement: Supplementary Figure 3 — STRING analysis of phosphorylated proteins enriched and statistically regulated in both treatment groups. The DMSO- and neratinib-enriched phosphorylated proteins, and the statistically regulated phosphorylated proteins, were combined into one dataset and analysed in STRING. A selection of the biological processes that were identified as statistically enriched are highlighted in colour. Although not statistically enriched, a number of proteins are also involved in the regulation of apoptosis (pink outlines). Lines indicate interactions between proteins; some proteins have multiple interactions, whereas others have none. [file Image_3.tif]

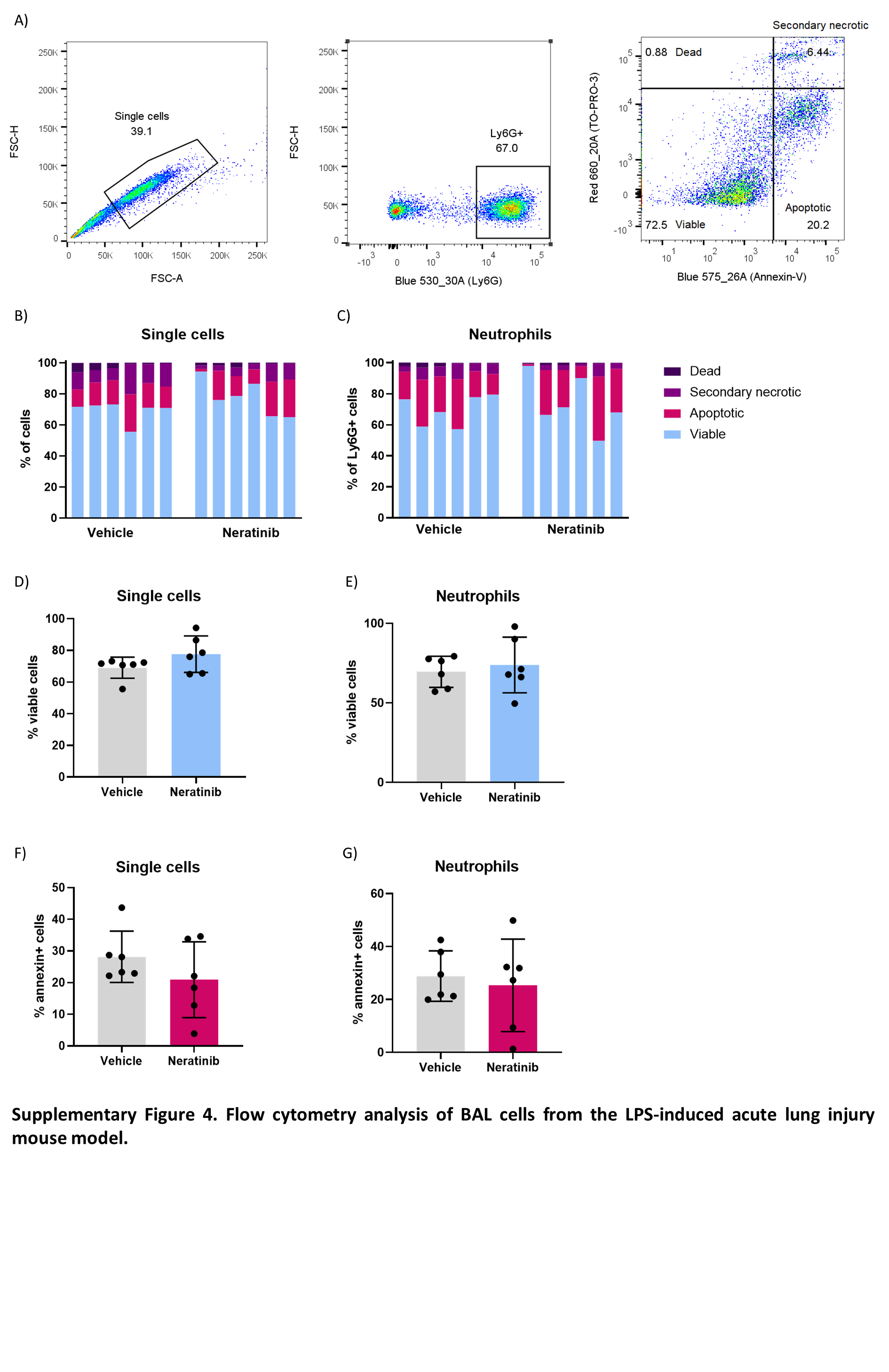

Supplement: Supplementary Figure 4 — Flow cytometry analysis of BAL cells from the LPS-induced acute lung injury mouse model. Cells in BAL were analyzed by flow cytometry. Single cells were initially gated (A, left panel), followed by Ly6G+ cells to identify neutrophils (A, middle panel). Annexin-V and TO-PRO-3 staining were then used to categorize cells as viable (negative for Annexin-V and TO-PRO-3), apoptotic (Annexin-V+), dead (TO-PRO-3+) and secondary necrotic (Annexin-V+ and TO-PRO-3+) (A, right panel). The single cells (B) and neutrophils (C) from each mouse BAL sample were categorized as such. Each bar represents data from one mouse. The percentage of viable cells (D) and viable neutrophils (E) was unchanged between treatment groups, as was the percentage of apoptotic cells (F) and apoptotic neutrophils (G). (D–G): each data point represents data from one mouse; bars show mean ± standard deviation. Unpaired t tests used for statistical analysis; no significant differences found between vehicle and neratinib treatment groups. [file Image_4.tif]
